# Three New Species of Shoot Fly, *Atherigona* spp., from Northern Thailand

**DOI:** 10.1673/031.011.13901

**Published:** 2011-10-20

**Authors:** Kittikhun Moophayak, Hiromu Kurahashi, Kabkaew L. Sukontason

**Affiliations:** ^1^ Department of Parasitology, Faculty of Medicine, Chiang Mai University, Chiang Mai 50200, Thailand; ^2^ Department of Medical Entomology, National Institute of Infectious Diseases, Tokyo 162-8640, Japan

**Keywords:** *Atherigona komi*, *Atherigona chiangmaiensis*, *Atherigona thailandica*, new species, shoot flies

## Abstract

Three new species of shoot fly, *Atherigona* Rondani (subgenus *Acritochaeta* Grimshaw) (Diptera: Muscidae), are described from northern Thailand, based on morphological characteristics of males. Unique features of *A. komi* sp. n. include a distinct spiral groove on the dorsal aspect of the fore femur and two dark apical wing spots, whereas *A. chiangmaiensis* sp. n. is recognized by the presence of one large patch on the apical wing spot, appearing as a large and smaller wave-shaped patch, and no distinct pattern on tergites. *A. thailandica* sp. n. displays a remarkable dark boomerang-shaped patch along the wing margin and fore femur, with two rows of long hairs on the dorsal surface. Male terminalia are also different in the new species, showing distinctive characteristics. This paper also presents five newly recorded species in Thailand; *Atherigona maculigera* Stein, *Atherigona ovatipennis vietnamensis* Shinonaga et Thinh, *Atherigona pallidipalpis* Malloch, *Atherigona seticauda* Malloch, and *Atherigona setitarsus* Shinonaga et Thinh. A key is provided for the adult males of *Atherigona* recorded in Thailand, all belonging to the subgenus *Acritochaeta*, except for *A. soccata* Rondani.

## Introduction

The shoot flies, *Atherigona* Rondani (Diptera: Muscidae), are small Diptera of the Muscidae family that are widely distributed in Old World tropical regions. Taxonomy of these flies has been investigated in Nepal (Pont 1972; Shinonaga and Singh 1994), the Philippines (Pont and Magpayo 1995), Vietnam (Shinonaga and Thinh 2000), and Borneo Island (Shinonaga 2009), but little investigative work has been done in Thailand. Sepsawadi et al. (1971) investigated the control of *Atherigona soccata* Rondani (subgenus *Atherigona*), which is the most economically important pest of sorghum. Pont and Magpayo (1995) studied the subgenus *Acritochaeta* Grimshaw, whose larvae act as scavenger or predators of decaying organic matter. *Atherigona orientalis* Schiner, a mechanical carrier of helminthic eggs and larvae, was reported in Brazil (de Oliveira et al. 2002); research conducted in China included *A. orientalis*, *A. simplex* (Thomson) and *Atherigona* sp. in the list of flies frequently associated with carcasses, suggesting possible forensic importance (Shi et al. 2009).

A large number of flies in the genus *Atherigona* were collected during the course of a faunistic study of flies with medical and/or forensic importance in Northern Thailand during 2009–2010; 1-day-old rotten spoiled beef viscera was used as bait. Using keys for identification (Shinonaga and Singh 1994; Pont and Magpayo 1995; Shinonaga and Thinh 2000), these collections revealed three species that had never been described, belonging to the subgenus *Acritochaeta.* These three species are new to science, based on the morphological difference of males from previous investigations. Furthermore, five species of *Atherigona* were recorded for the first time in Thailand. The primary aim of this paper is to describe three new species based on the peculiar characteristics of males. For comprehensive comparison among all species of *Atherigona* recorded in Thailand, unique characteristics and male terminalia of all available species, along with a key for male identification, have been included.

## Materials and Methods

The fly specimens described herein as a new species were collected in 2009–2010. One-day-old rotten spoiled beef viscera (300g) were used as bait, and sweeping nets and traps (30 × 30 × 50 cm) were used to capture individuals. The beef viscera were left at room temperature (∼ 25–30 °C) 24 hours before use. After identification using keys of Shinonaga and Singh (1994), Pont and Magpayo (1995), and Shinonaga and Thinh (2000), these species were identified as new to science. Males were examined under a dissecting microscope (Olympus, www.olympusglobal.com) to identify external morphological features such as the wing, leg, and abdomen. Photographs of the whole body, wing, and abdomen were taken with a Nikon E-800 (Nikon, www.nikon.com), and Adobe Photoshop CS3 was used to adjust the brightness and contrast of the images.

To examine the terminalia, the last abdominal segment of the specimens that had already been taken for photographs was dissected under a dissecting microscope (Olympus) using fine forceps. To clear the integument, 10% potassium hydroxide solution with one drop of 70% ethanol was used to soak the specimens for 24 hours before a thorough examination. The terminalia were transferred onto a microscopic slides containing a few drops of 99% glycerol. The illustration was performed from the identical features under a compound microscope (Olympus). Terminology of the adult morphology follows McAlpine (1981), and male terminalia follows Sinclair (2000). Specimen depositories are cited using the following abbreviation: NSMT, National Science Museum, Tokyo.

**Figure 1.**
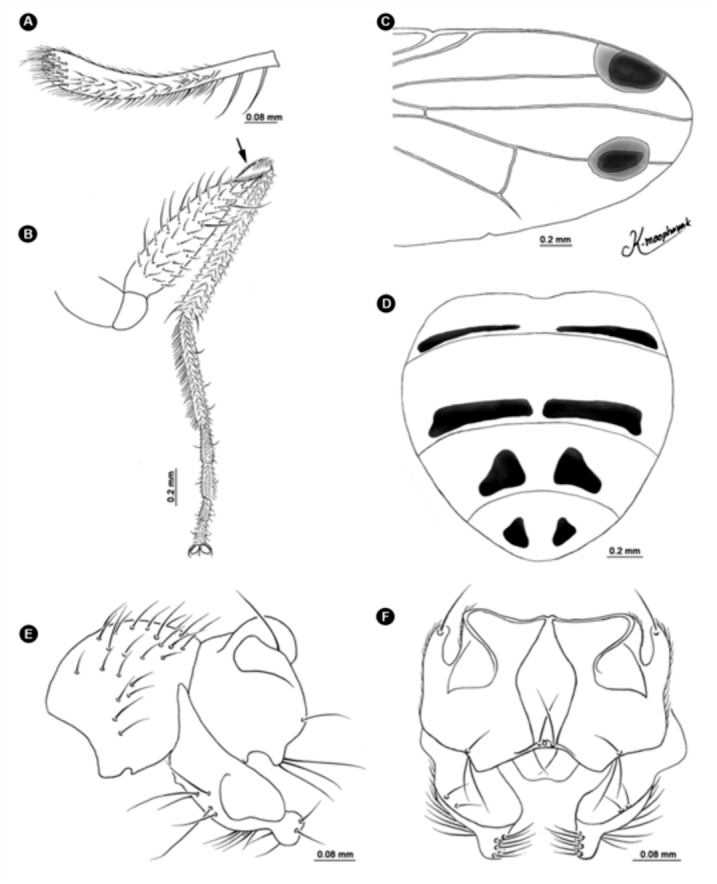
*Atherigona komi* sp. nov. (A) Palpus, left lateral view; (B) fore femur with spiral groove (arrow); (C) wing; (D) abdomen, dorsal view; (E) male terminalia, left lateral view; (F) male terminalia, dorsal view. High quality figures are available online.

**Figure 2.**
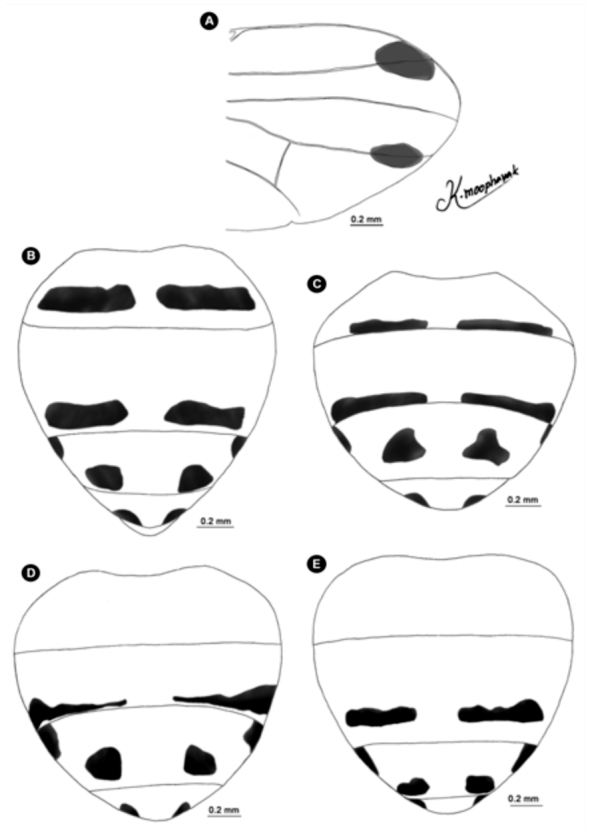
Variation of *Atherigona komi* sp. nov. (A) Wing; (B, C) abdomen with a pair of well-separated, dark brown marginal bands at tergite 1+2, dorsal view; (D, E) abdomen without a pair of well-separated, dark brown marginal bands at tergite 1+2, dorsal view. High quality figures are available online.

**Figure 3.**
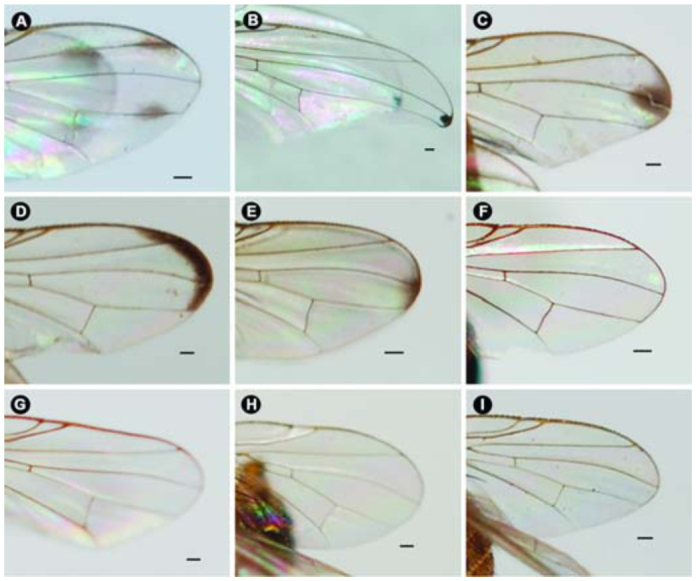
Wing of *Atherigona.* (A) *A. komi* sp. nov.; (B) *A. ovatipennis vietnamensis*; (C) *A. chiangmaiensis* sp. nov.; (D) *A. thailandica* sp. nov.; (E) *A. setitarsus*; (F) *A. seticauda*; (G) *A. maculigera*; (H) *A. pallidipalpis*; (I) *A. orientalis.* Scale bar = 0.2 mm. High quality figures are available online.

**Figure 4.**
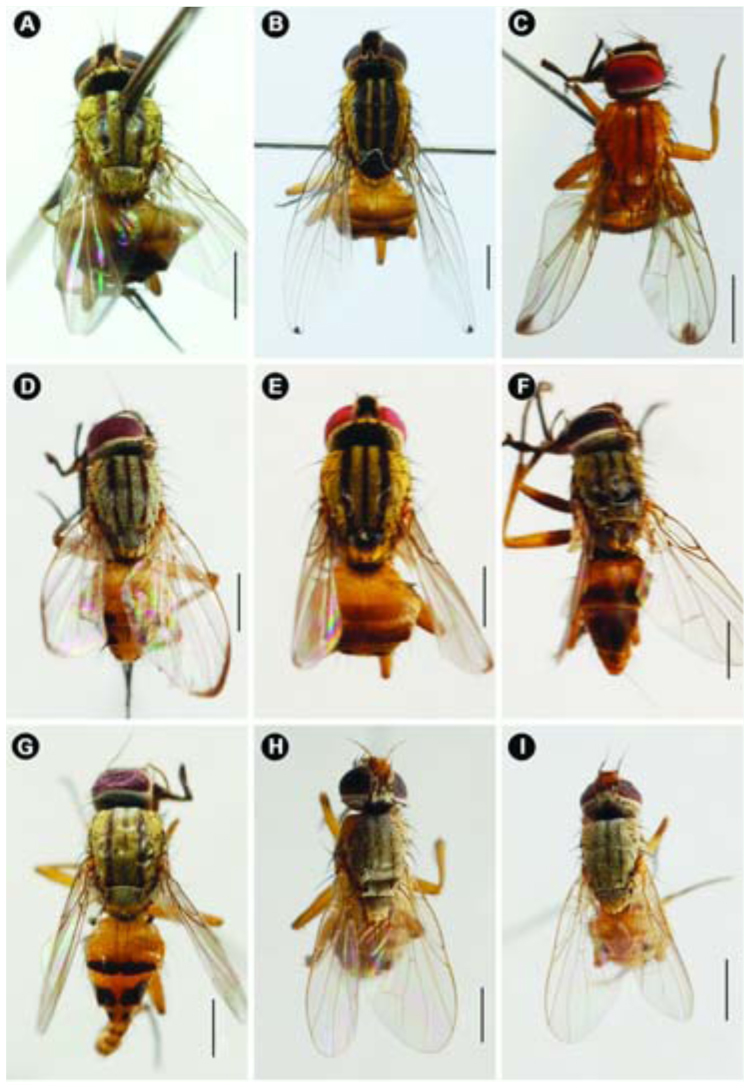
Habitus photographs of *Atherigona*, dorsal view. (A) *A. komi* sp. nov.; (B) *A. ovatipennis vietnamensis*; (C) *A. chiangmaiensis* sp. nov.; (D) *A. thailandica* sp. nov.; (E) *A. setitarsus;* (F) *A. seticauda*; (G) *A. maculigera*; (H) *A. pallidipalpis;* (I) *A. orientalis.* Scale bar = 1 mm. High quality figures are available online.

**Figure 5.**
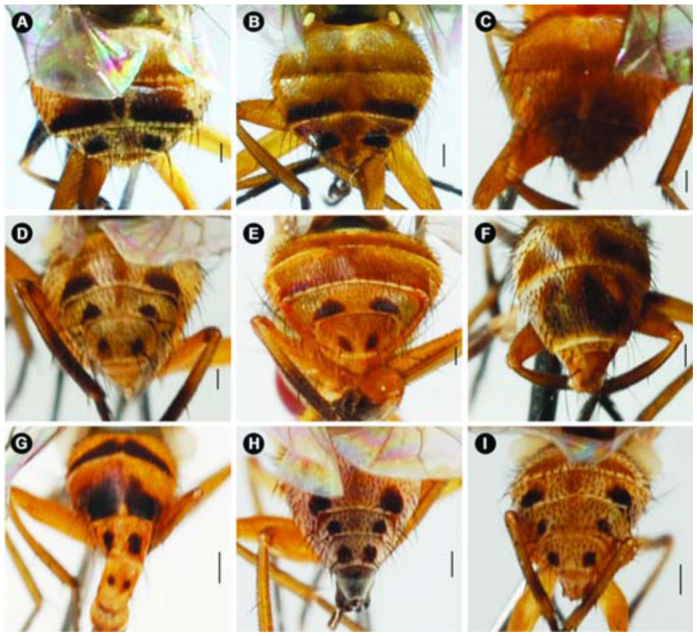
Abdomen *of Atherigona*, dorsal view. (A) *A. komi* sp. nov.; (B). *A. ovatipennis vietnamensis*; (C) *A. chiangmaiensis* sp. nov.; (D) *A. thailandica* sp. nov.; (E) *A. setitarsus*; (F) *A. seticauda*; (G) *A. maculigera*; (H) *A. pallidipalpis*; (I) *A. orientalis.* Scale bar = 0.2 mm. High quality figures are available online.

**Figure 6.**
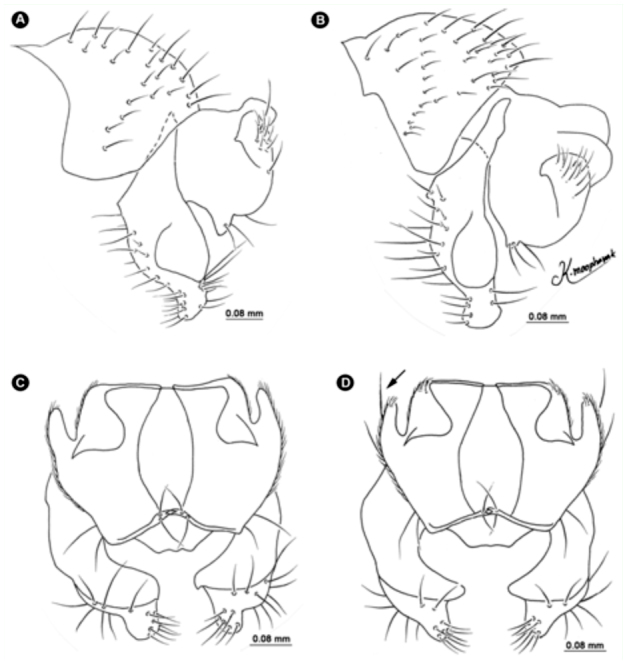
Variation of *Atherigona komi* sp. nov. (A, B) Male terminalia, left lateral view; (C) male terminalia without long distinct seta on cercal plate, dorsal view; (D) male terminalia with long distinct seta (arrow) on cercal plate, dorsal view. High quality figures are available online.

**Figure 7.**
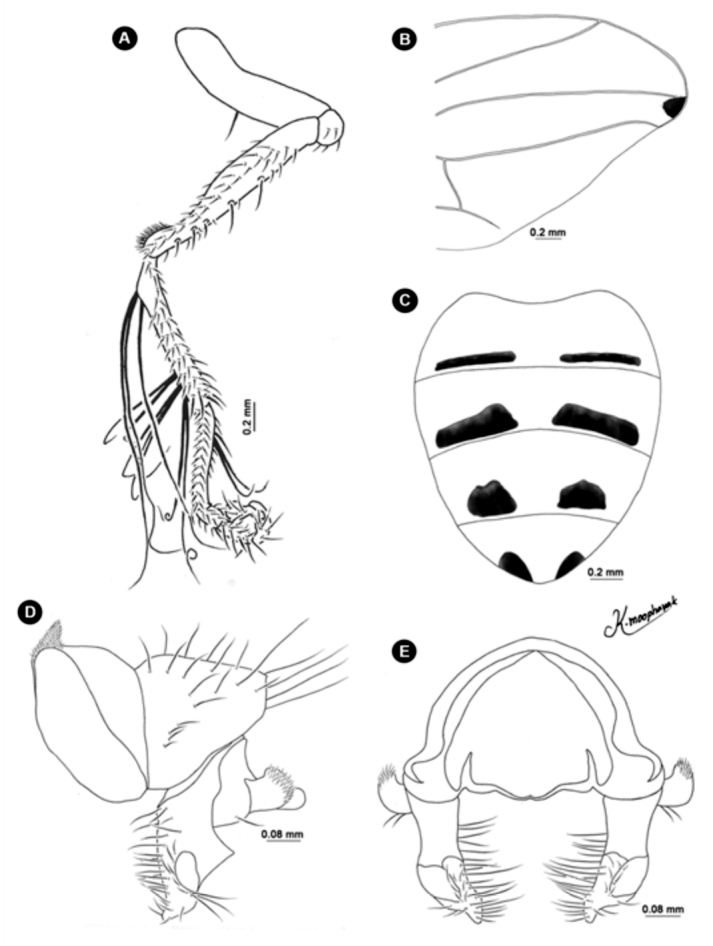
*Atherigona ovatipennis vietnamensis.* (A) Fore femur, tibia, and tarsus. Three long bristles on basal part and nine long bristles on distal ⅓ that curled on apices of tibia. (B) wing; (C) abdomen, dorsal view; (D) male terminalia, left lateral view; (E) male terminalia, dorsal view. High quality figures are available online.

**Figure 8.**
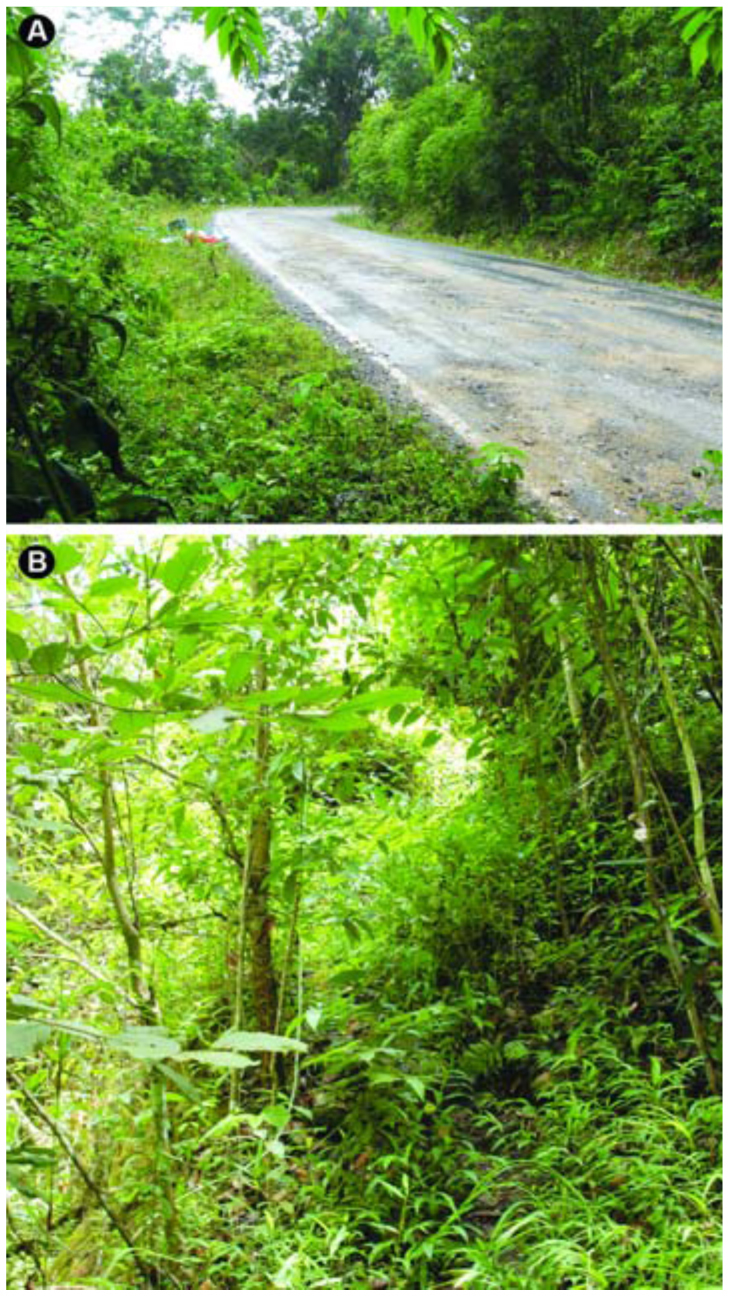
The biotopes of Chiang Mai province, northern Thailand. (A) Locality of the bush with high tree along the local highway where the holotypes of *Atherigona komi* sp. nov. and *Atherigona thailandica* sp. nov. were collected; (B) locality of the dense forest (1142 m altitude), about 200 m away from the local highway where the holotype of *Atherigona chiangmaiensis* sp. nov. was collected. High quality figures are available online.

***Atherigona* (*Acritochaeta*) *komi* sp. n. (Figures 1, 2, 3A, 4A, 5A, 6)**
Diagnosis
  Because of its elongate palpus (Figure 1A), lack of hypopygial prominence, and lack of a trifoliate process at end of the epandrium, this new species was grouped in the subgenus *Acritochaeta.* Distinctive traits of the male of *Atherigona komi* include a distinct spiral groove on the dorsal aspect of the fore femur (Figure 1B) and two dark apical wing spots (Figures 1C, 2A, 3A). This new species is closely related to *A. ovatipennis vietnamensis* Shinonaga et Thinh reported from Vietnam (Shinonaga and Thinh 2000) by the similarity of abdominal pattern on all tergites (Figure 5B). However, distinctive characteristics used to differentiate between these two similar species are the (1) two large dark wing spots located at vein R_2+3_ and M in *A. komi* (Figures 1C, 2A, 3A), while only one small dark spot located between vein R_2+3_ and M is seen in *A. ovatipennis vietnamensis* (Figures 3B, 7B), the (2) fore femur; the presence of spiral groove on dorsal surface in *A. komi* (Figure 1B) but absence in *A. ovatipennis vietnamensis*, the (3) fore tibia; the presence of long bristles curled at apices on basal and distal parts in *A. ovatipennis vietnamensis* (Figure 7A), but absence in *A. komi* (Figure 1B), and the (4) fore tarsus; the enlarged pulvillus and empodium in *A. ovatipennis vietnamensis* (Figure 7A), but normally sized in *A. komi* (Figure 1B).
Description of holotype male
**Body length.** 4.13 mm (range 3.30–4.62 mm), from 13 specimens including holotype (Figure 4A). Wing-length 3.10 mm (range 2.70–3.44 mm).
**Head.** Eyes bare and dichoptic. Frontal vitta black with orange apical ⅖, in two specimens with dark orange, in one specimen with wholly black (Table 1). Fronto-orbital plate grey pollinose. Antennal scape, pedicel, and postpedicel entirely black. Arista pubescent dark brown with light brown at basal ⅖. Palpus wholly black, elongated with slight dilation at apex, with fine hairs along apical ventral part and two strong hairs anteroventrally (Figure 1A).
**Thorax.** Ground-color grey dusted with three broad black longitudinal stripes (Figure 4A). Apex of scutellum orange; postpronotal lobe yellow; anterior and posterior spiracles yellow; basal lateral scutellar setae almost half as long as subbasal lateral setae. Chaetotaxy of scutum: *acr* in 3 or 4 rows at suture; *dc* 0+5-6; *ial* 1+1; *pprn* 2; *npl* 2; *kepst* 3. Leg with orange coxae and trochanters; fore femur black with orange at base and apex, with spiral groove on dorsal surface (Figure 1B); fore tibia and tarsomere black, only orange on tibia basal ⅕; mid femur orange, with 1 or 2 *p* (Table 1); mid tibia orange, dark on apical half, with 1 *p*; mid tarsomere entirely black; hind femur entirely orange; hind tibia black with orange on basal ⅕, with 1 *ad*, 1 *av*, 1 *pd*; hind tarsomere entirely black. Wing hyaline (Figure 3A), with 2 apical dark spots located at veins R2+3 and M (Figures 1C, 2A, 3A); vein M slightly bending anteriorly; cross vein r-m slightly before middle of cell dm. Lower calypter yellowish-white; upper calypter yellowish-white with white below. Knob of halteres white.
**Abdomen.** Abdomen ground-color orange (Figure 5A). Tergite 1+2 with a pair of well-separated, dark brown marginal bands (Figures 1D, 2B, 2C, 5A), though a few specimens did not have dark brown marginal bands (Figures 2D, 2E) (Table 1); tergite 3 with a pair of well-separated, broad dark brown marginal bands, ¼ – ½ tergal length (Figures 1D, 2B–E, 5A); tergite 4 with a pair of well-separated, dark brown subtriangular spots, ¾ tergal length (Figures 1D, 2B–E, 5A); tergite 5 with a pair of well-separated, dark brown subtriangular spots, ⅓ tergal length (Figures 1D, 2B–E).
**Terminalia.** No hypopygial prominence observed at the proximal epandrium and trifoliate process at distal epandrium (Figures 1E, 6A, 6B); cercal plate, on extreme upper margin, with (Figures 1F, 6D) or without a long distinct setae (Figure 6C).
**Female.** Unknown.
**Holotype**
  ♂, Thailand: Chiang Mai, Mae Rim, Mae Raem, Tardmok waterfall (18°57′34″N, 98°50′06″E), 805 m, 23-IX-2009, K. Moophayak. Holotype (NSMT-I-Dip6787) deposited in NSMT.
**Paratypes**
  6♂, same data as holotype. 1♂, Thailand: Chiang Mai, Muang, Suthep-Pui Mt. (18° 48′21″ N, 98° 54′ 39″ E), 1104 m, 13-XI-2009, K. Moophayak; 1♂, Thailand: Chiang Mai, Muang, Suthep-Pui Mt. (18° 48′ 20″ N, 98° 54′ 34″ E), 950 m, 6-IX-2009, T. Klongklaew; 1♂, Thailand: Phitsanulok, Wang Thong, Huai Nam Phong (16° 51′ 53″ N, 100° 31′ 01″ E), 252 m, 20-IX-2009, N. Bunchu; 3♂, Thailand: Phitsanulok, Wang Thong, Huai Nam Phong (16° 5′ 53″ N, 100° 31′ 01″ E), 252 m, 22-IX-2009, N. Bunchu; 2♂, Thailand: Chiang Mai, Muang, Suthep-Pui Mt. (18° 48′ 21″ N, 98° 54′ 39″ E), 1104 m, 22-IX-2010, K. Moophayak; 3♂, Thailand: Chiang Mai, Mae Rim, Tardmok waterfall (18° 57′ 34′ N, 98° 50′ 06″ E), 805 m, 21-IX-2010, K. Moophayak.
**Remarks**
  This species was collected in traps (30 × 30 × 50 cm) with 300 g of one-day-old rotten spoiled beef viscera, not one-day-old rotten spoiled mackerel.
**Distribution**
  Known only from the type locality, Chiang Mai and Phitsanulok provinces of Thailand.
**Bionomics**
  Adult males were collected using one-day-old rotten spoiled beef viscera or one-day-old rotten spoiled pork viscera as bait, at altitudes ranging from 805-1104 m. The collection site was the bush with the high tree along the local highway (Figure 8A). This species was collected from the late rainy season (September) to early winter (November).
**Etymology**
  This new species was named in honor of Dr. Kom Sukontason for his support and encouragement of fly research in Thailand.

**Table 1.**
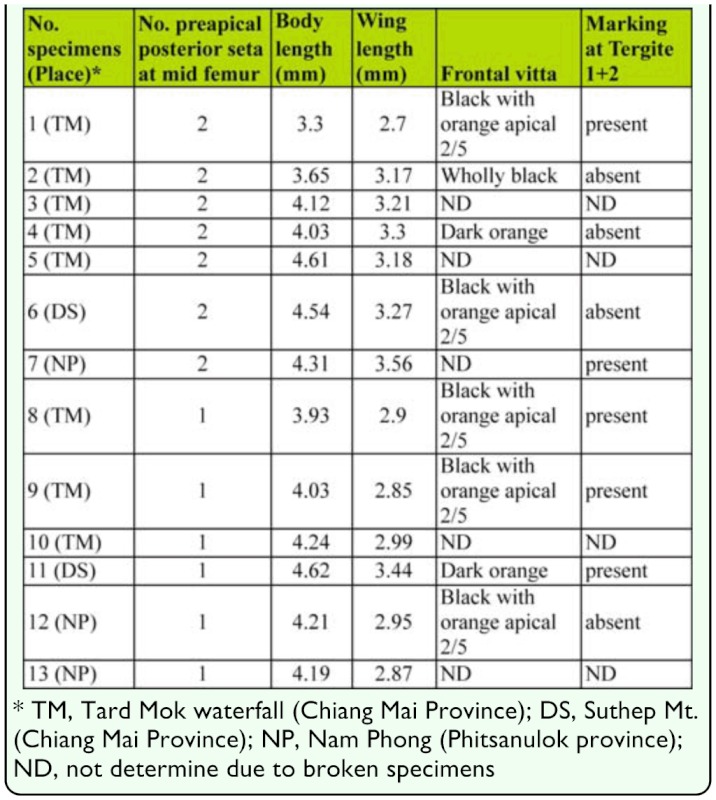
Intraspecific variation of characteristics observed in *Atherigona komi* sp. n.

***Atherigona* (*Acritochaeta*) *ovatipennis vietnamensis* Shinonaga et Thinh (Figures 3B, 4B, 5B, 7A–E)**
**Specimens examined**
  1♂, Thailand: Chiang Mai, Chom Thong (18° 30′ 52″ N, 98° 31′ 33″ E), 1494 m, 11-XI-2009, K. Moophayak.
**Distribution**
  Vietnam (Shinonaga and Thinh 2000) and Thailand (*new record*). The collection site in Thailand was the high forest area, altitude 1494 m.

**Figure 9.**
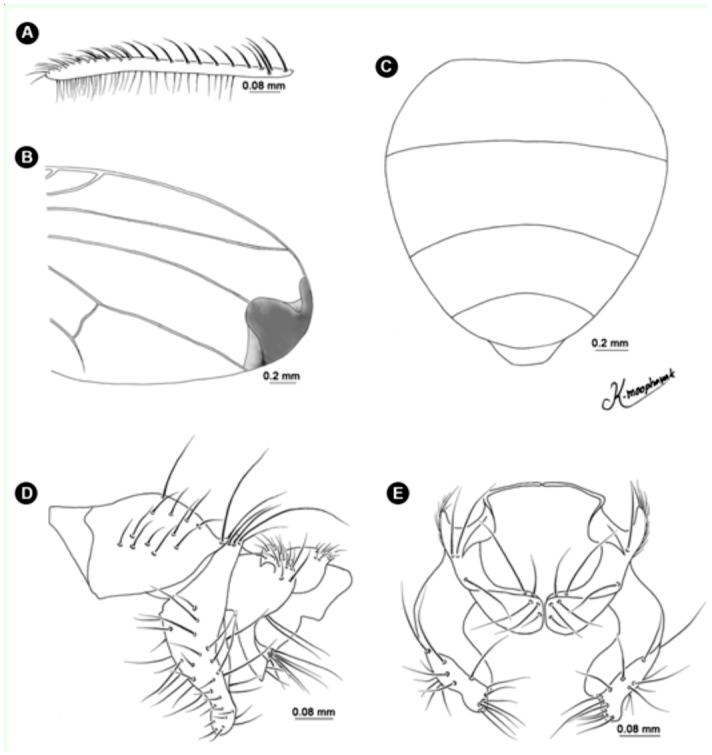
*Atherigona chiangmaiensis* sp. nov. (A) Palpus, left lateral view; (B) wing; (C) abdomen, dorsal view; (D) male terminalia, left lateral view; (E) male terminalia, dorsal view. High quality figures are available online.

***Atherigona* (*Acritochaeta*) *chiangmaiensis* sp. n. (Figures 3C, 4C, 5C, 9A–E)**
**Diagnosis**.
  This new species was placed in the subgenus *Acritochaeta* for having an elongate palpus (Figure 9A); presence of dark apical wing-spot (Figure 3C); no hypopygial prominence; and without a trifoliate process at the end of the epandrium. Males of *A. chiangmaiensis* can be recognized by the presence of one large patch on the apical wing spot (Figure 3C) appearing as a large and smaller wave-shaped patch (Figure 9B), an orange body (Figure 4C), and no distinct pattern on tergites (Figures 5C, 9C).
**Description of holotype male**
**Body length.** 5.27 mm (1 specimen) (Figure 4C). Wing-length 3.27 mm.
**Head.** Eyes bare and dichoptic; frontal vitta orange that is basally dark; fronto-orbital plate grey pollinose. Antennal scape and pedicel orange; postpedicel black; arista pubescent, black with orange at basal ⅖*.* Palpus wholly black, elongated with slight dilation at apex, with fine hairs along ventral part and strong hairs along dorsal part (Figure 9A).
**Thorax.** Entirely ground-color orange with one narrowed dark longitudinal median vitta (Figure 4C); postpronotal lobe yellow; anterior and posterior spiracles yellow; basal lateral scutellar setae almost half as long as subbasal lateral setae. Chaetotaxy of scutum: *acr* in 4 or 5 rows at suture; *dc* 0+5-6; *ial* 1+1; *pprn* 1; *npl* 2; *kepst* 3. Leg entirely orange; fore femur with excavation on dorsal surface; mid femur orange with 2 *p*; mid tibia with 1 *p*; hind tibia black with 1 *ad*, 1 *av*, 1 *pd.* Wing hyaline (Figure 3C) with one large dark spot from midway between veins R_2+3_ and R_4+5_ to just beyond vein M (Figures 3C, 9B); M slightly bending anteriorly; cross vein r-m slightly before middle of cell dm. Lower and upper calypters clear yellow. Knob of halteres pale yellow.
**Abdomen.** Ground-color orange. Tergite 1+2 without any bands or patterns marginally (Figures 5C, 9C).
**Terminalia.** Without hypopygial prominence at proximal epandrium and trifoliate process at distal epandrium (Figure 9D); cercal plate, on sub-upper margin, with one or two long distinct setae (Figure 9E).
**Female.** Unknown.
**Holotype**
  1♂, Thailand: Chiang Mai, Doi Saket, Doi Nang Kaew (19° 03′ 53″ N, 99° 22′ 34″ E), 1142 m, 14-XI-2009, K. Moophayak. Holotype (NSMT-I-Dip 6788) deposited in NSMT.
**Paratypes**
  1♂, Thailand: Chiang Mai, Muang, Suthep-Pui Mt. (18° 48′ 21″ N, 98° 54′ 39″ E), 1104 m, 22-IX-2010, K. Moophayak; 3♂, Thailand: Chiang Mai, Mae Rim, Tardmok waterfall (18° 57′ 34″ N, 98° 50′ 06″ E), 805 m, 21-IX-2010, K. Moophayak.
**Taxonomic Discussion**
  *A. chiangmaiensis* sp. n. is a member of the subgenus *Acritochaeta* based on the presence of a dark apical wing-spot, an elongate palpus, lack of a hypopygial prominence, and lack of a trifoliate process at the end of the epandrium. This new species is closely related to *A. gigantipunctata* Shinonaga reported from Vietnam (Shinonaga and Thinh 2000) by the similarity of the abdominal pattern, which lacks any patterns or marginal bands, as well as the similarity of one large dark spot from midway between veins R_2+3_ and R_4+5_ to just beyond vein M. However, the shape of this wing spot is markedly different between these two species; a large and smaller wave-shaped patch is seen in *A. chiangmaiensis*, compared to a vertical oval patch seen in *A. gigantipunctata.*
**Distribution**
  Known only from the type locality, Chiang Mai province, Thailand.
**Bionomics**
  Adult males were collected using one-day-old rotten spoiled beef viscera or one-day-old rotten spoiled pork viscera as bait, at an altitude of 1142 m. Specimens were unable to be collected using one-day-old rotten spoiled mackerel. The collected site was the dense forest ∼ 200 m away from the local highway (Figure 8B). This species was collected only in the late rainy season (September) to early winter (November).
**Etymology**
  The new species epithet refers to Chiang Mai province, in which the type of material of the new species was collected.

**Figure 10.**
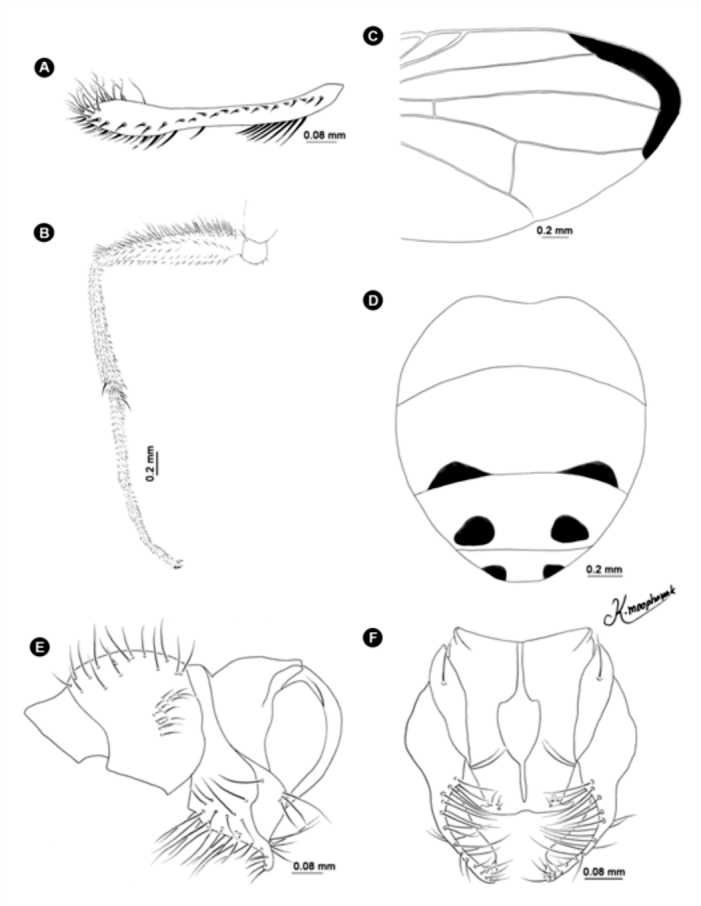
*Atherigona thailandica* sp. nov. (A) palpus, left lateral view; (B) fore femur; (C) wing; (D) abdomen, dorsal view; (E) male terminalia, left lateral view; (F) male terminalia, dorsal view. High quality figures are available online.

***Atherigona* (*Acritochaeta*) *thailandica* sp. n. (Figures 3D, 4D, 5D, 10A–E)**
**Diagnosis**
  This new species is placed in the subgenus *Acritochaeta* for having an elongate palpus (Figure 10A), the presence of a dark apical wing-spot (Figures 3D, 10C), lack of a hypopygial prominence, and lack of a trifoliate process at the end of the epandrium. Males of *A. thailandica* can be recognized by the presence of a dark boomerang-shaped patch along the margin, extending from the distal ⅓ between veins R_1_, R_2+3_ to just below vein M (Figures 3D, 10C), cross vein r-m at the middle of cell dm, and fore femur with two rows of long hairs on the dorsal surface.
**Description of holotype male**
**Body length.** 3.87 mm (range 3.56–4.22 mm) (8 specimens including holotype) (Figure 4D). Wing-length 3.23 mm (range 3.02–3.52 mm).
**Head.** Eye bare and dichoptic, frontal vitta black, fronto-orbital plate grey pollinose. Antennal scape, pedicel, and postpedicel entirely black; arista pubescent, dark brown with orange on basal ⅗*.* Palpus wholly black, elongated with slight dilation at apex (Figure 10A).
**Thorax.** Ground-color grey with three black longitudinal stripes of the same width (Figure 4D); apex of scutellum orange; postpronotal lobe yellow; anterior and posterior spiracle yellow; basal lateral scutellar setae almost half as long as subbasal lateral setae. Chaetotaxy of scutum: *acr* in 3 or 4 rows at suture; *dc* 0+5-6; *ial* 1+1; *pprn* 1; *npl* 2; *kepst* 3. Leg with orange coxae and trochanters; foreleg almost completely black, except orange at basal ⅕ – ⅗ of femur and on basal ⅖ of tibia; fore femur with two rows of long hairs on dorsal surface; mid leg entirely orange; mid femur entirely orange, with 2 *p*; hind leg almost orange, except darker at tip and ½ femur and tibia, respectively; hind tibia with 1 *ad*, 1 *av*, 1 *pd.* Wing hyaline with dark boomerang-shaped patch along the wing margin, extending from distal ⅓ between veins R_1_ and R_2+3_ to just below vein M (Figures 3D, 10C); M slightly bending anteriorly; cross vein r-m at the middle of cell dm. Lower and upper calypters yellowish-white. Knob of halteres white.
**Abdomen.** Ground-color yellow; tergite 1+2 with indistinct marginal band; tergite 3 with indistinct median vitta and a pair of small dark brown marginal bands, ⅖ – ⅗ tergal length (Figures 5D, 10D); tergite 4 with a pair of well-separated, dark brown more or less triangular spots, ⅓ – ⅓ tergal length (Figures 5D, 10D); tergite 5 with a pair of smaller dark brown round spots, ^⅓^ tergal length (Figures 5D, 10D).
**Terminalia.** Lacking hypopygial prominence at proximal epandrium and trifoliate process at distal epandrium; surstylus with distinct line of hairs along inner margin (Figures 10E, 10F).
**Female.** Unknown.
Holotype
  1♂, Thailand: Chiang Mai, Mae Rim, Tardmok waterfall (18° 57′ 34″ N, 98° 50′ 06″ E), 805 m, 23-IX-2009, K. Moophayak. Holotype (NSMT-I-Dip 6789) deposited in NSMT.
**Paratypes**
  1♂, same data as holotype; 2♂, Thailand: Chiang Mai, Muang, Suthep-Pui Mt. (18° 48′ 21″ N, 98° 54′ 34″ E), 1104 m, 13-XI-2009, K. Moophayak; 3♂, Thailand: Chiang Mai, Doi Saket, Doi Nang Kaew (19° 03′ 53″ N, 99° 22′ 34″ E), 1142 m, 14-XI-2009, K. Moophayak; 1♂, Thailand: Chiang Mai, Doi Saket, Doi Nang Kaew (19° 03′ 53″ N, 99° 22′ 34″ E), 1142 m, 14-XI-2009, K.L. Sukontason; 1♂, Thailand: Chiang Mai, Doi Saket, Doi Nang Kaew (19° 03′ 53″ N, 99° 22′ 34″ E), 1142 m, 14-XI-2009, T. Klongklaew; 1♂, Thailand: Chiang Mai, Mae Rim, Tardmok waterfall (18° 57′ 34″ N, 98° 50′ 06″ E), 805 m, 21-IX-2010, K. Moophayak.
**Taxonomic Discussion**
  *A. thailandica* can be distinguished from the other species of the subgenus *Acritochaeta* for having unique wing features; a dark boomerang-shaped patch along the wing margin, extending from distal *Vi* between veins R1 and R2+3 to just below vein M. The other characteristics are the position of a cross vein, of which r-m at the middle of cell dm and fore femur with two rows of long hairs on dorsal surface.
**Bionomics**
  Adult males were collected using one-day-old rotten spoiled beef viscera as bait, at altitudes ranging from 805-1104 m. However, they could not be collected using one-day-old rotten spoiled pork viscera or one-day-old rotten spoiled mackerel as bait. The collection site was the bush with the high tree along the local highway (Figure 8A). This species was collected only in the late rainy season (September) to early winter (November).
**Distribution**
  Known only from the type locality, Chiang Mai province, Thailand.
**Etymology**
  The species epithet refers to Thailand, where the type material of the new species was collected.

**Figure 11.**
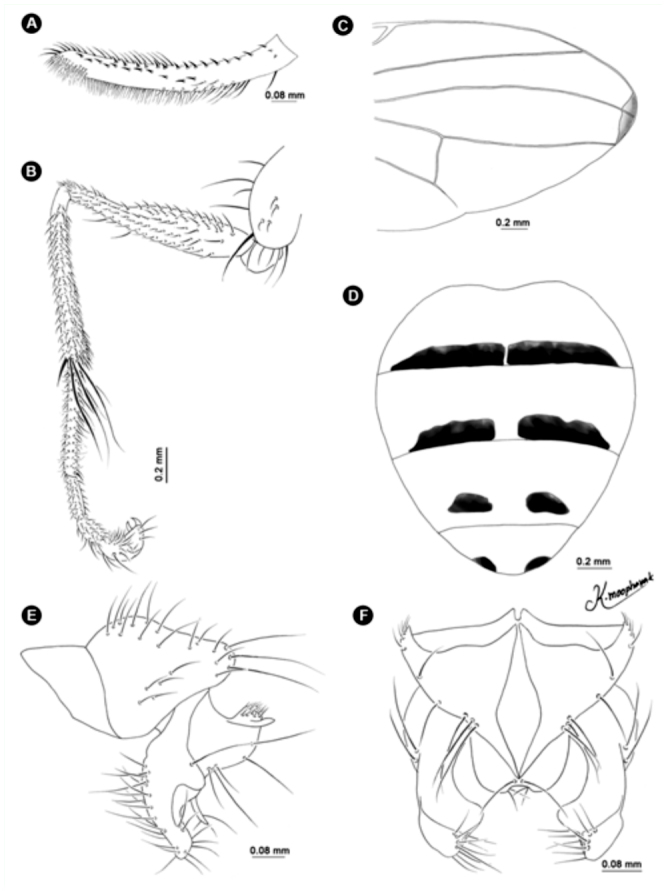
*Atherigona setitarsus.* (A) Palpus, left lateral view; (B) fore femur; (C) wing; (D) abdomen, dorsal view; (E) male terminalia, left lateral view; (F) male terminalia, dorsal view. High quality figures are available online.

***Atherigona* (*Acritochaeta*) *setitarsus* Shinonaga et Thinh (Figures 3E, 4E, 5E, 11A–F)**
**Species examined**
  1♂, Thailand: Chiang Mai, Mae Rim, Tardmok waterfall (18° 57′ 34″ N, 98° 50′ 06″ E), 805 m, 23-IX-2009, K. Moophayak; 2♂, Thailand: Chiang Mai, Muang, Suthep-Pui Mt. (18° 48′ 21″ N, 98° 54′ 34″ E), 1104 m, 25-IX-2009, R. Ngoen-klan; 2♂, Thailand: Chiang Mai, Doi Saket, Doi Nang Kaew (19° 03′ 53″ N, 99° 22′ 34″ E), 1142 m, 14-XI-2009, K.L. Sukontason; 2♂, Thailand: Chiang Mai, Doi Saket, Doi Nang Kaew (19° 03′ 53″ N, 99° 22′ 34″ E), 1142 m, 14-XI-2009, K. Moophayak; 1♂, Thailand: Chiang Mai, Chom Thong (18° 30′ 52″ N, 58° 31′ 33″ E), 1494 m, 11-XI-2009, T. Klongklaew.
**Distribution**
  Vietnam (Shinonaga and Thinh 2000) and Thailand (*new record*). The collection sites in Thailand were in an urban area, altitude ranging from 805–1494 m.

**Figure 12.**
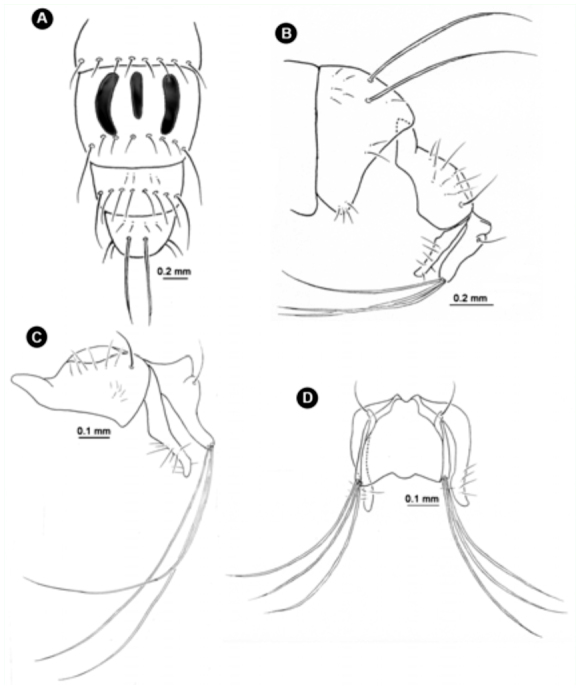
*Atherigona seticauda.* (A) Abdomen, dorsal view; (B) male terminalia focusing on the long caudal setae on tergite 5, left lateral view; (C) male terminalia of other specimen after removing tergite 5, left lateral view; (D) male terminalia, dorsal view. High quality figures are available online.

***Atherigona (Acritochaeta) seticauda* Malloch (Figures 3F, 4F, 5F, 12A–D)**
**Specimens examined**
  1♂, Thailand: Chiang Mai, Doi Saket, Doi Nang Kaew (19° 03′ 53″ N, 99° 22′ 34″ E), 1142 m, 14-XI-2009, R. Ngoen-klan; 1♂, Thailand: Chiang Mai, Doi Saket, Doi Nang Kaew (19° 03′ 53″ N, 99° 22′ 34″ E), 1142 m, 14-XI-2009, T. Klongklaew; 2♂, Thailand: Chiang Mai, Hang Dong (18° 47′ 20″ N, 98° 50′ 28″ E), 499 m, 20-V-2009, K. Moophayak; 1♂, Thailand: Chiang Mai, Hang Dong (18° 47′ 20″ N, 98° 50′ 28″ E), 499 m, 9-VI-2009, K. Moophayak; 2♂, Thailand: Chiang Mai, Hang Dong (18° 47′ 20″ N, 98° 50′ 28″ E), 499 m, 13-VIII-2009, K. Moophayak; 2♂, Thailand: Chiang Mai, Hang Dong (18° 47′ 20″ N, 98° 50′ 28″ E), 499 m, 1-IX-2009, K. Moophayak; 4♂, Thailand: Chiang Mai, Mae Rim, Tardmok waterfall (18° 57′ 34″ N, 98° 50′ 06″ E), 805 m, 3-VII-2009, K. Moophayak; 2♂, Thailand: Chiang Mai, Mae Rim, Tardmok waterfall (18° 57′ 34″ N, 98° 50′ 06″ E), 805 m, 8-IX-2009, K. Moophayak; 5♂, Thailand: Chiang Mai, Mae Rim, Pong Yaeng (18° 53′ 14″ N, 98° 49′ 53″ E), 750 m, 8-IV-2009, K. Moophayak; 10♂, Thailand: Chiang Mai, Mae Rim, Pong Yaeng (18° 53′ 14″ N, 98° 49′ 53″ E), 750 m, 21-V-2009, K. Moophayak; 2♂, Thailand: Chiang Mai, Mae Rim, Pong Yaeng (18° 53′ 14″ N, 98° 49′ 53″ E), 750 m, 14-VIII-2009, K. Moophayak; 1♂, Thailand: Chiang Mai, Mae Rim, Pong Yaeng (18° 53′ 14″ N, 98° 49′ 53″ E), 750 m, 2-IX-2009, K. Moophayak; 1♂, Thailand: Chiang Mai, Mae Rim, Pong Yaeng (18° 55′ 36″ N, 98° 54′ 09″ E), 357 m, 2-IX-2009, K. Moophayak; 1♂, Thailand: Chiang Mai, Muang (18° 46′ 51″ N, 99° 57′ 10″ E), 349 m, 21-IX-2009, K. Moophayak.
**Distribution**
  Malaysia, Sri Lanka, Sumatra, Philippines (Pont and Magpayo 1995), and Thailand (*new record*). The collection sites in Thailand were in an urban area, altitude ranging from 349–1142 m.

**Figure 13.**
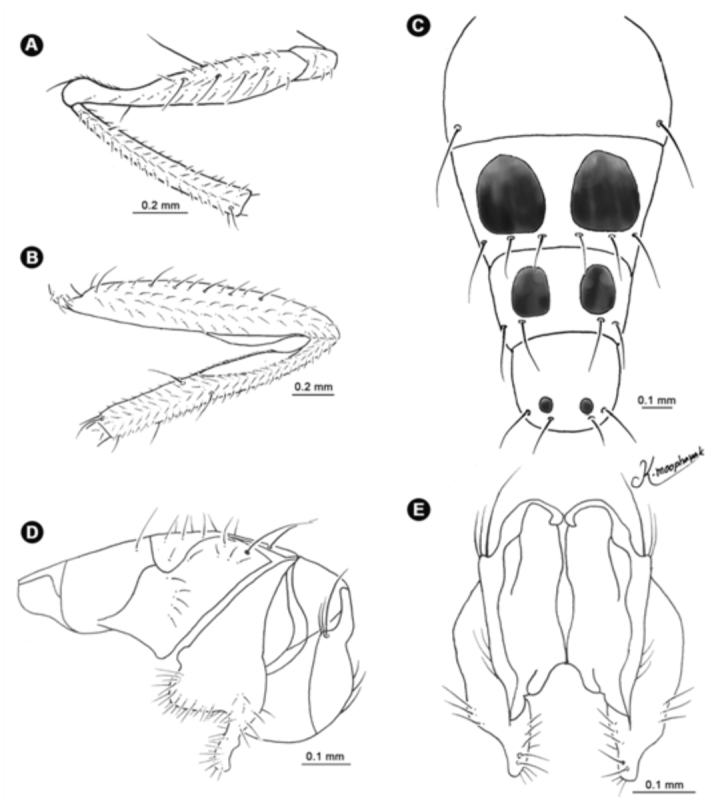
*Atherigona maculigera.* (A) Fore leg showing femur and tibia; (B) hind leg showing femur and tibia; (C) abdomen, dorsal view; (D) male terminalia, left lateral view; (E) male terminalia, dorsal view. High quality figures are available online.

***Atherigona (Acritochaeta) maculigera* Stein (Figures 3G, 4G, 5G, 13A–E)**
**Specimens examined**
  2♂, Thailand: Chiang Mai, Doi Saket, Doi Nang Kaew (19° 03′ 53″ N, 99° 22′ 34″ E), 1142 m, 14-XI-2009, K.L. Sukontason; 3♂, Thailand: Chiang Mai, Doi Saket, Doi Nang Kaew (19° 03′ 53″ N, 99° 22′ 34″ E), 1142 m, 14-XI-2009, K. Moophayak; 3♂, Thailand: Chiang Mai, Doi Saket, Doi Nang Kaew (19° 03′ 53″ N, 99° 22′ 34″ E), 1142 m, 14-XI-2009, R. Ngoen-klan; 2♂, Thailand: Chiang Mai, Doi Saket, Doi Nang Kaew (19° 03′ 53″ N, 99° 22′ 34″ E), 1142 m, 14-XI-2009, T. Klongklaew; 2♂, Thailand: Chiang Mai, Mae Rim, Tardmok waterfall (18° 57′ 34″ N, 98° 50′ 06″ E), 805 m, 8-IV-2009, K. Moophayak; 2♂, Thailand: Chiang Mai, Mae Rim, Tardmok waterfall (18° 57′ 34″ N, 98° 50′ 06″ E), 805 m, 3-VII-2009, K. Moophayak; 1♂, Thailand: Chiang Mai, Mae Rim, Tardmok waterfall (18° 57′ 34″ N, 98° 50′ 06″ E), 805 m, 23-IX-2009, K. Moophayak; 1♂, Thailand: Chiang Mai, Mae Rim, Pong Yaeng (18° 53′ 14″ N, 98° 49′ 53″ E), 750 m, 8-IV-2009, K. Moophayak; 1♂, Thailand: Chiang Mai, Mae Rim, Pong Yaeng (18° 53′ 14″ N, 98° 49′ 53″ E), 750 m, 2-IX-2009, K. Moophayak; 1♂, Thailand: Chiang Mai, Muang (18° 55′ 40″ N, 98° 57′ 18″ E), 334 m, 26-V-2009, K. Moophayak; 5♂, Thailand: Chiang Mai, Muang, Suthep-Pui Mt. (18° 47′ 19″ N, 98° 55′ 15″ E), 817 m, 15-III-2008, K. Moophayak; 1♂, Thailand: Chiang Mai, Muang, Suthep-Pui Mt. (18° 47′ 19″ N, 98° 55′ 15″ E), 950 m, 6-IX-2009, R. Ngoen-klan; 2♂, Thailand: Chiang Mai, Muang, Suthep-Pui Mt. (18° 47′ 19″ N, 98° 55′ 15″ E), 950 m, 6-IX-2009, T. Klongklaew; 3♂, Thailand: Chiang Mai, Muang, Suthep-Pui Mt. (18° 47′ 19″ N, 98° 55′ 15″ E), 950 m, 25-IX-2009, R. Ngoen-klan; 2♂, Thailand: Chiang Mai, Muang, Suthep-Pui Mt. (18° 48′ 21″ N, 98° 54′ 39″ E), 1104 m, 13-XI-2009, K. Moophayak; 4♂, Thailand: Chiang Mai, Muang, Suthep-Pui Mt. (18° 48′ 19″ N, 98° 54′ 37″ E), 1138 m, 9-IV-2009, K. Moophayak; 2♂, Thailand: Chiang Mai, Muang, Suthep-Pui Mt. (18° 48′ 19″ N, 98° 54′ 37″ E), 1138 m, 25-V-2009, K. Moophayak.
**Distribution**
  Philippines, Malaysia, Sri Lanka, Sulawesi, Sumatra, Taiwan (Pont and Magpayo 1995), and Thailand (*new record*). The collection sites in Thailand were in an urban area, altitude ranging from 334–1142 m.

**Figure 14.**
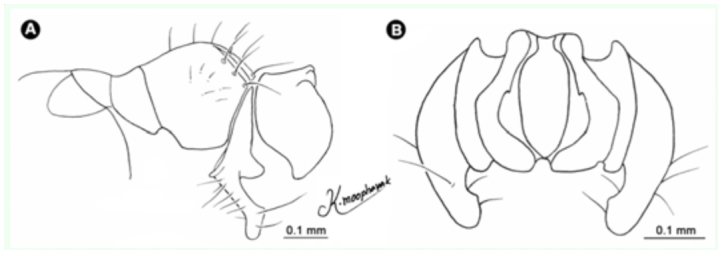
*Atherigona pallidipalpis.* (A) Male terminalia, left lateral view; (B) male terminalia, dorsal view. High quality figures are available online.

***Atherigona (Acritochaeta) pallidipalpis* Malloch (Figures 3H, 4H, 5H, 14A,B)**
**Specimens examined**
  2♂, Thailand: Chiang Mai, Doi Saket, Doi Nang Kaew (19° 03′ 53″ N, 99° 22′ 34″ E), 1142 m, 14-XI-2009, R. Ngoen-klan; 1♂, Thailand: Chiang Mai, Hang Dong (18° 41′ 21″ N, 98° 59′ 09″ E), 294 m, 30-VI-2009, K. Moophayak; 3♂, Thailand: Chiang Mai, Mae Rim, Tardmok waterfall (18° 57′ 34″ N, 98° 50′ 06″ E), 805 m, 8-IV-2009, K. Moophayak; 1♂, Thailand: Chiang Mai, Mae Rim, Tardmok waterfall (18° 57′ 34″ N, 98° 50′ 06″ E), 805 m, 3-VII-2009, K. Moophayak; 1♂, Thailand: Chiang Mai, Mae Rim, Pong Yaeng (18° 55′ 36″ N, 98° 54′ 09″ E), 357 m, 14-VIII-2009, K. Moophayak; I♂, Thailand: Chiang Mai, Mae Rim, Pong Yaeng (18° 55′ 36″ N, 98° 54′ 09″ E), 357 m, 2-IX-2009, K. Moophayak; 1♂, Thailand: Chiang Mai, Mae Rim, Pong Yaeng (18° 53′ 14″ N, 98° 49′ 53″ E), 750 m, 8-IV-2009, K. Moophayak; 1♂, Thailand: Chiang Mai, Mae Rim, Pong Yaeng (18° 53′ 14″ N, 98° 49′ 53″ E), 750 m, 3-VII-2009, K. Moophayak; 1♂, Thailand: Chiang Mai, Mae Rim, Pong Yaeng (18° 53′ 14″ N, 98° 49′ 53″ E), 750 m, 21-VII-2009, K. Moophayak; 1♂, Thailand: Chiang Mai, Mae Rim, Pong Yaeng (18° 53′ 14″ N, 98° 49′ 53″ E), 750 m, 2-IX-2009, K. Moophayak; 1♂, Thailand: Chiang Mai, Muang (18° 45′ 22″ N, 98° 55′ 21″ E), 8-VI-2009, K. Moophayak; 1♂, Thailand: Chiang Mai, Muang (18° 46′ 44″ N, 99° 04′ 48″ E), 284 m, 26-VII-2009, K. Moophayak.
**Distribution**
  India, Malaysia, Indonesia, Philippines, Melanesia? (Pont and Magpayo 1995), and Thailand (*new record*). The collection sites in Thailand were in an urban area, altitude ranging from 284–1142 m.

**Figure 15.**
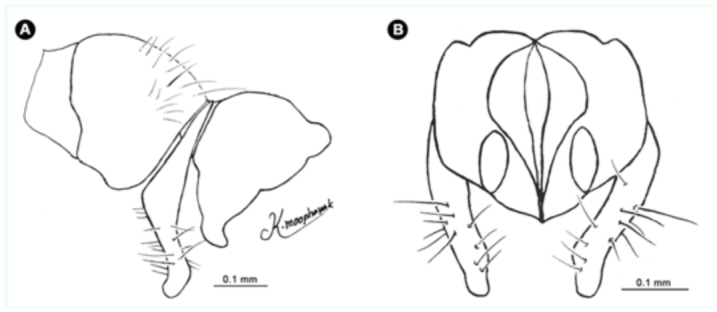
*Atherigona orientalis.* (A) Male terminalia, left lateral view; (B) male terminalia, dorsal view. High quality figures are available online.

***Atherigona (Acritochaeta) orientalis* Schiner (Figures 3I, 4I, 5I, 15A, 15B)**
**Specimens examined**
  1♂, Thailand: Chiang Mai, Hang Dong (18° 41′ 21″ N, 98° 59′ 09″ E), 294 m, 26-VII-2009, K. Moophayak; 1♂, Thailand: Chiang Mai, Hang Dong (18° 42′ 30″ N, 98° 55′ 57″ E), 5-IX-2009, K. Moophayak; 1♂, Thailand: Chiang Mai, Hang Dong (18° 42′ 30″ N, 98° 55′ 57″ E), 22-X-2009, K. Moophayak; 1♂, Thailand: Chiang Mai, Hang Dong (18° 47′ 20″ N, 98° 50′ 28″ E), 499 m, 20-V-2009, K. Moophayak; 1♂, Thailand: Chiang Mai, Hang Dong (18° 44′ 14″ N, 98° 59′ 09″ E), 372 m, 18-V-2009, K. Moophayak; 1♂, Thailand: Chiang Mai, Hang Dong (18° 44′ 14″ N, 98° 59′ 09″ E), 372 m, 8-VI-2009, K. Moophayak; 1♂, Thailand: Chiang Mai, Hang Dong (18° 45′ 29″ N, 98° 52′ 07″ E), 378 m, 1-IV-2009, K. Moophayak; 1♂, Thailand: Chiang Mai, Hang Dong (18° 45′ 29″ N, 98° 52′ 07″ E), 378 m, 20-V-2009, K. Moophayak; 1♂, Thailand: Chiang Mai, Hang Dong (18° 45′ 29″ N, 98° 52′ 07″ E), 378 m, 9-VI-2009, K. Moophayak; 28♂, Thailand: Chiang Mai, Mae Rim, Tardmok waterfall (18° 57′ 34″ N, 98° 50′ 06″ E), 805 m, 8-IV-2009, K. Moophayak; 8♂, Thailand: Chiang Mai, Mae Rim, Tardmok waterfall (18° 57′ 34″ N, 98° 50′ 06″ E), 805 m, 27-V-2009, K. Moophayak; 1♂, Thailand: Chiang Mai, Mae Rim, Saluang (19° 07′ 48″ N, 98° 54′ 34″ E), 353 m, 16-VIII-2009, K. Moophayak; 5♂, Thailand: Chiang Mai, Mae Rim, Pong Yaeng (18° 55′ 36″ N, 98° 54′ 09″ E), 357 m, 21-V-2009, K. Moophayak; 2♂, Thailand: Chiang Mai, Mae Rim, Pong Yaeng (18° 55′ 36″ N, 98° 54′ 09″ E), 357 m, 16-VI-2009, K. Moophayak; 2♂, Thailand: Chiang Mai, Mae Rim, Pong Yaeng (18° 53′ 14″ N, 98° 49′ 53″ E), 750 m, 21-V-2009, K. Moophayak; 1♂, Thailand: Chiang Mai, Mae Rim, Pong Yaeng (18° 53′ 14″ N, 98° 49′ 53″ E), 750 m, 3-VII-2009, K. Moophayak; 1♂, Thailand: Chiang Mai, Mae Rim (18° 51′ 34″ N, 98° 32′ 51″ E), 331 m, 10-VI-2009, K. Moophayak; 1♂, Thailand: Chiang Mai, Mae Rim (18° 51′ 34″ N, 98° 32′ 51″ E), 331 m, 16-VIII-2009, K. Moophayak; 2♂, Thailand: Chiang Mai, Muang (18° 49′ 22″ N, 99° 04′ 12″ E), 305 m, 7-VII-2009, K. Moophayak; 2♂, Thailand: Chiang Mai, Muang (18° 55′ 40″ N, 98° 57′ 18″ E), 334 m, 8-VII-2009, K. Moophayak; 1♂, Thailand: Chiang Mai, Muang (18° 46′ 44″ N, 99° 04′ 48″ E), 284 m, 26-VII-2009, K. Moophayak; 1♂, Thailand: Chiang Mai, Muang (18° 45′ 22″ N, 98° 55′ 21″ E), 10-VIII-2009, K. Moophayak; 1♂, Thailand: Chiang Mai, Muang (18° 45′ 22″ N, 98° 55′ 21″ E), 1-IX-2009, K. Moophayak; 1♂, Thailand: Chiang Mai, Muang (18° 46′ 51″ N, 99° 57′ 10″ E), 349 m, 19-V-2009, K. Moophayak; 1♂, Thailand: Chiang Mai, Muang (18° 46′ 51″ N, 99° 57′ 10″ E), 349 m, 9-VI-2009, K. Moophayak.
**Distribution**
  Cosmotropical including Thailand. The collection sites in Thailand were in an urban area, altitude ranging from 284–805 m.

***Atherigona (Atherigona) soccata* Rondani**
No specimens were collected in this study; however, there was a record for this fly control in Thailand (Sepsawadi et al. 1971).
**Distribution**
  Pakistan, India, Nepal, Myanmar, China (Guangdong), Philippines, Thailand, southern Europe, North Africa, Middle East, throughout the Afrotropical region (Pont and Magpayo 1995)

**Key to the species of *Atheriogona* recorded in Thailand (only for males)**
1. Palpus elongate, slightly dilated at apex. Abdomen without hypopygial prominence and trifoliate process subgenus *Acritochaeta* 2
Palpus short, dilated at apex. Abdomen with hypopygial prominence and trifoliate process Subgenus *Atherigona* (Thailand only *A. soccata* recorded)
2. Wing with apical dark spot or band or patch 3
Wing wholly clear 7
3. Wing with 2 apical dark spots located at R_2+3_ and M. Fore femur with spiral groove *A. komi* sp. n.
Wing with one band or patch along margin 4
4. Tergites without any band and pattern *A. chiangmaiensis* sp. n.
Tergites with marginal band or pattern, at least T3, T4 5
5. Fore tibia and tarsomere without long setae. Fore femur almost black with two rows of long hairs on dorsal surface. Wing with dark boomerang-shaped patch along the wing margin, extending from distal ⅓ between veins R_1_ and R_2+3_ to just below vein M *A. Thailandica* sp. n.
Fore tibia and/or tarsomere with long setae 6
6. Fore tibia with three long setae distally. Fore tarsomere with numerous long hairs. Pulvilli and empodium of fore tarsus normal, not enlarged. Wing with dark narrow vertical patch along margin from midway between veins R_2+3_ and R_4+5_ to just beyond vein M *A. setitarsus*
Fore tibia with long curled setae basally and distally. Fore tarsomere without long hairs. Pulvilli and empodium of fore tarsus enlarged. Wing with dark narrow spot located between veins R_4+5_ and M *A. ovatipennis vietnamensis*
7. Tergite 5 with one pair of long stout setae. Three pairs of long stout setae at lower margin of cercus *A. seticauda*
Tergite 5 without of any long stout setae 8
8. Hind femur and tibia with ventral keel *A. maculigera*
Hind femur and tibia without ventral keel 9
9. Fore tarsomere with fine hairs *A. pallidipalpis*
Fore tarsomere without fine hairs *A. orientalis*

## Abbreviations:

**NSMT**,: National Science Museum, Tokyo;
***acr***,: presutural acrostichal setae;
***ad***,: anterodorsal seta;
***kepst***,: katepisternal setae;
***av***,: anteroventral seta;
***dc***,: dorsocentral setae;
***ial***,: intra-alar setae;
***npl***,: notopleural setae;
***p***,: preapical posterior seta;
***pd***,: posterodorsal seta;
***pprn***,: postpronotal setae
